# Digital Twin-Assisted Lightpath Provisioning and Nonlinear Mitigation in C+L+S Multiband Optical Networks †

**DOI:** 10.3390/s24248054

**Published:** 2024-12-17

**Authors:** Sadegh Ghasrizadeh, Prasunika Khare, Nelson Costa, Marc Ruiz, Antonio Napoli, Joao Pedro, Luis Velasco

**Affiliations:** 1 Advanced Broadband Communications Center (CCABA), Universitat Politècnica de Catalunya (UPC), 08034 Barcelona, Spain; mohammadsadegh.ghasrizadeh@upc.edu (S.G.); prasunika.khare@upc.edu (P.K.); marc.ruiz-ramirez@upc.edu (M.R.); 2 Infinera Unipessoal Lda., Carnaxide, 2790-078 Oeiras, Portugal; ncosta@infinera.com (N.C.); jpedro@infinera.com (J.P.); 3 Infinera, 81541 Munich, Germany; 4 Instituto de Telecomunicações, 1049-001 Lisbon, Portugal

**Keywords:** multiband optical transmission, digital twin, quality of transmission, service provisioning, nonlinear mitigation

## Abstract

Multiband (MB) optical transmission targets increasing the capacity of operators’ optical transport networks. However, nonlinear impairments (NLI) affect each optical channel in the C+L+S bands differently, and, therefore, the routing and spectrum assignment (RSA) problem needs to be complemented with fast and accurate tools to consider the quality of transmission (QoT) within the provisioning process. This paper proposes a digital twin-assisted approach for lightpath provisioning to provide a complete solution for the RSA problem that ensures the required QoT in MB optical networks. The OCATA time domain digital twin is proposed, not only to estimate the QoT of a selected path but also to support the QoT-based channel assignment process. OCATA is based on a Deep Neural Network (DNN) to model the propagation of the optical signal. However, because of the different impacts of nonlinear noise on each channel and the large number of channels that need to be considered in C+L+S MB scenarios, OCATA needs to be adapted to make it scalable, while keeping its high accuracy and fast QoT estimation characteristics. In consequence, a complete methodology is proposed in this work that limits the number of channels being modeled to just a few. Moreover, OCATA-MB helps to mitigate NLI noise by programming the receiver at the provisioning time and thus with very little complexity compared to its equivalent implemented during the operation. NLI noise mitigation can be applied in the case when a lightpath cannot be provisioned because none of the available channels can provide the required QoT, making it an advantageous tool for reducing connection blocking. Exhaustive simulation results demonstrate the remarkable accuracy of OCATA-MB in estimating the QoT for any channel. Interestingly, by utilizing the proposed OCATA-MB-assisted lightpath provisioning approach, a reduction of the blocking ratio exceeding 50% when compared to traditional approaches is shown when NLI noise mitigation is not applied. If NLI mitigation is implemented, an additional over 50% blocking reduction is achieved.

## 1. Introduction

Optical transport networks play a vital role in coping with the ever-increasing capacity demand fostered by beyond 5G (B5G) technology. To address future network demands and emerging B5G services, the ITU-T recently launched a focus group to study the capabilities of networks and establish a road map toward the year 2030 [1]. In this regard, multiband (MB) optical networks are seen as a solution to extend legacy optical networks’ capacity [2]. The authors in [3] analyzed the expected traffic demand to be supported as a result of combining well-known mass market services with foreseen B5G services scenarios and showed that MB transmission will be required for all network segments.

However, the adoption of MB optical transmission requires dealing with a phenomenon that increases nonlinear impairments (NLI)—Inter-channel Stimulated Raman Scattering (ISRS) [4]. Several studies have analyzed and assessed the increment of capacity achievable by using other bands, e.g., the S-band, in addition to the traditional C and L ones, from the perspective of the line system [5]. However, in addition to pure optical transmission, the provisioning of optical connections needs to be reviewed also to actually achieve the expected capacity increase.

Traditionally, the provisioning of optical connections entails solving the Routing and Spectrum Allocation (RSA) problem [6], which can be complemented by considering the quality of transmission (QoT) of the computed route and the spectral allocation before accepting the connection request (see, e.g., [7,8,9,10]). Estimation of the QoT has been thoroughly investigated, and several techniques have been proposed, e.g., based on the Gaussian Noise model [11,12]. However, the larger impact of the ISRS effect on MB transmission and the large number of signals being transmitted increase the complexity of estimating the QoT. This in turn demands solutions that can provide similar accuracy but at lower computational complexity [13,14].

Due to the high complexity and limited time available for QoT estimation during the provisioning phase, the application of Machine Learning (ML) techniques [15] can provide significant advantages [16]. In addition, ML models can be used to construct a digital twin (DT) of the optical network [17]. In fact, the use of a DT is seen as an essential tool for network automation and zero-touch networking. The authors of [18,19,20,21] proposed OCATA, a DT for the optical time domain based on the application of Deep Neural Networks (DNN) modeling optical components, and experimentally showed that it can be used as a reliable and low-complex approach for accurately estimating the QoT, specifically the pre-Forward Error Correction (FEC) Bit Error Rate (BER), of an optical connection.

The main drawback to using ML techniques is the availability of large datasets for training the models. This is especially important in the case of training DNNs. In the case of optical transmission, the datasets can be generated from experiments and from simulation. However, few experimental data are available for MB optical transmission, and simulation tools require a long running time to produce data as a result of the large number of channels that need to be simulated. For that very reason, the authors in [22] proposed a method for solving the generalized nonlinear Schrödinger equation that runs much faster than the traditional split-step Fourier method, thus providing an efficient and accurate method for large training dataset generation.

In addition to QoT estimation, recent advancements in Digital Signal Processing (DSP) and coherent detection make it possible to configure optical transponders to operate with different sets of transmission parameters, named operational modes, including the modulation format (MF), symbol rate (SR), and FEC [23]. The authors in [24] proposed a complete workflow for connection provisioning that includes selection of the operational mode that better fits the candidate lightpath. This, however, can result in the set-up of several lightpaths that together provide the required capacity [25].

Some other works in the literature have proposed nonlinear mitigation techniques to improve the QoT by adapting the detection areas in the receiver (Rx) using ML techniques (see e.g., [26]). Such approaches are difficult to implement in practice, since the algorithms need to run in the DSP itself.

In our previous paper [27], we proposed using the OCATA DT to accurately estimate the pre-FEC BER of the different channels during lightpath provisioning, as well as to mitigate the NLI noise in the Rx by optimizing the detection areas of the in-phase (I) and quadrature (Q) constellation points of the higher-order quadrature amplitude modulation (QAM) optical signals. Note that the proposed nonlinear mitigation is computed at provisioning time, so the DSP uses the optimized detection areas as a simple map.

In this paper, we extend [27] and present a complete methodology to adapt OCATA for MB optical transmission and provide the needed support to the RSA algorithm in charge of the selection of a route and channel assignment running in the software-defined networking (SDN) controller. Specifically, the contributions of this paper are:

- The OCATA-MB methodology sketched in Section 2, which enables the application of digital twinning solutions for accurate, fast, and scalable QoT estimation and nonlinear mitigation. Such applications allow the DT to be part of the lightpath provisioning process in the SDN controller, in particular for channel assignment.
- The models and algorithmic approaches to implement the OCATA-MB methodology and the on-line MB-RSA, described in Section 3. The proposed algorithms include: (*i*) selection of the reference channels to be used for the DNN link modeling within the OCATA-MB; (*ii*) composition of the features for the non-reference channels from those of the reference ones; (*iii*) computation of the optimal detection areas to implement nonlinear mitigation in the Rx; and (*iv*) a general OCATA-assisted on-line MB-RSA algorithm for lightpath provisioning and OCATA-based channel selection.

The discussion is supported by the exhaustive simulation results presented in Section 4. Finally, Section 5 concludes the paper.

## 2. Provisioning MB Optical Connections

Lightpath provisioning is one of the most important problems that needs to be addressed to automate network operation. Lightpath provisioning includes the selection of a route and a channel assignment to ensure that the required QoT, e.g., in terms of the pre-FEC BER, is met. In case the required QoT cannot be assured, the lightpath request is rejected, i.e., blocked.

In this section, we first present the MB scenario considered in this paper and provide observations that will be used afterwards for improving lightpath provisioning. Next, the OCATA time domain digital twin is introduced, as it includes precise models trained for different channels. To facilitate its application in MB optical transmission, a specific methodology is proposed. Finally, OCATA-MB is proposed as a method to mitigate the impact of the ISRS effect. Because OCATA can generate the expected optical constellations for an optical connection and a channel assignment, such an expected signal can be used to instruct the DSP on the Rx side, which can improve the QoT of the lightpath.

### 2.1. Considered MB Scenario and Channel Performance

**Figure 1:**
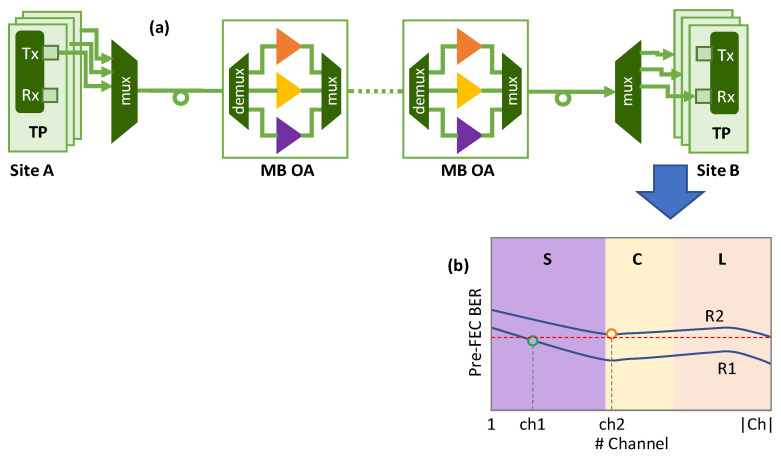
Overview of the considered MB scenario (**a**) and illustrative performance of MB optical transmission (**b**).

Figure 1a shows the considered MB scenario where a set of MB optical transponders (TPs) is available in sites A and B and can work in the different bands. The signals generated by the Txs are multiplexed and transmitted along the optical fiber, where MB Optical Amplifiers (OA) are employed, with the optical amplification structure consisting of using one OA per band, e.g., Erbium-Doped Fiber Amplifiers (EDFA) for the C and L bands and a Thulium-Doped Fiber Amplifier (TDFA) for the S-band, together with waveband (de)multiplexers.

As already introduced, by transferring power from higher to lower frequencies, the ISRS effect increases the impact of fiber nonlinearities, which impact the QoT of optical connections using the different channels in a nonidentical manner. To illustrate such a differentiated QoT, Figure 1b shows the pre-FEC BER that an optical connection using a given route on the optical network will experience when different channels are used. Two routes (R1 and R2) are considered with different total lengths, with R2 being longer than R1.

In general, we observe that the channels in the S-band experience a higher BER for the same route, and therefore they can support shorter optical connections, as compared with the channels in the C and L bands that can support longer connections. In view of the above, one approach for channel assignment could be to select channels in the C or L bands, as they are more versatile. However, this approach will result in faster exhaustion of the C and L bands, so new medium-to-long reach connection requests would get blocked with a higher probability. Another approach could be to find the channel that results in a BER that is high but under the threshold; e.g., channel *ch1* in the S-band is selected over route R1 in Figure 1b. This has the potential to minimize the probability that new optical connections are blocked. The main drawbacks of this approach are: (1) the QoT has to be estimated for all the available channels along the route, which can require a long computation time depending on the method used for such an estimation; and (2) it might happen that any available channel can provide a BER under the threshold, so the connection request is blocked; e.g., in R2, channel ch2 in the C-band provides the best BER, but that is above the threshold.

In the remainder of this section, we propose solutions to implement the second approach, i.e., to find the optimal channel assignment for a given route in an efficient way, as well as to devise mitigation methods to improve the QoT of those optical connections with channel assignments providing poor QoT that would otherwise be blocked.

### 2.2. OCATA for MB Optical Transmission

**Figure 2:**
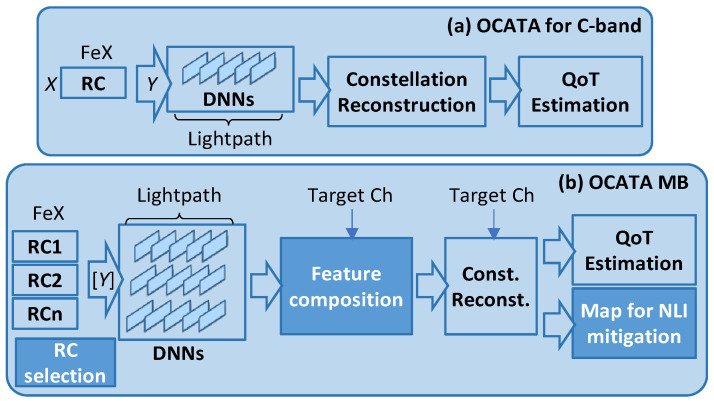
Main building blocks of the OCATA-MB time domain digital twin.

The classical OCATA architecture for the C-band [18,19,20] (see Figure 2a) considers DNN models that are independent of the specific channel; a reference channel (RCh) in the middle of the C-band is considered. Incoming optical signals generated using the modulation format *m*-QAM are processed in the time domain. Therefore, incoming in-phase (I) and quadrature (Q) optical constellations with *m* distinct constellation points (CP) are processed. Specifically, an input IQ optical constellation (*X*) consists of a set of symbols *x*∈*X*, where each *x* = [*x^I^, x^Q^*] belongs to a CP. A features extraction (FeX) module finds features for each CP of the received signal by applying Gaussian mixture model (GMM) fitting [28], which characterizes the CP as a set of bivariate Gaussian distributions. The output of the FeX module is a set of features *Y*, which contains five features for each CP, i.e., *Y^i^* = (*μ^I^*, *μ^Q^*, *σ^I^*, *σ^Q^*, *σ^IQ^*)*^i^* is the vector of features for CP *i*, where *μ^I^* and *μ^Q^* represent the mean position for *i* over the *I* and *Q* axes, respectively, *σ^I^* and *σ^Q^* are the variance of *i* over the axes, and *σ^IQ^* is the covariance between the axes. It is worth noting that the dispersion of symbols that belong to a certain CP provides valuable insight into the level of noise affecting the signal, both linear (LI) and NLI noise. As the total noise increases, dispersion of the symbols also increases. The FeX module is followed by a set of DNNs modeling the propagation of the signal on the optical components in the route of the lightpath. DNNs are pre-trained for the RCh, so the concatenated DNN representing the signal propagation can be very quickly composed by selecting individual DNN models from a repository. Such propagation increases the LI and NLI noise, resulting in higher values for *σ^I^* and *σ^Q^* at the output of the DNNs, which can be related to QoT-related indicators like pre-FEC BER and SNR [20]. For scalability reasons, the DNN models consider the propagation of the features for a subset of selected CPs only (*selCP*). Precisely, two exterior and two interior CPs are selected, as proposed in [19], and from them a Constellation Reconstruction block reconstructs the features for the non-propagated CPs with high accuracy, i.e.,

(1) $$\begin{array}{cc} {Y^{i}=F}^{i}\left(\left[Y^{j},\forall j\in selCP\right]\right) & \forall i\in CP \end{array}$$

The Constellation Reconstruction block can be implemented as a DNN that takes as input propagated features [*Y^j^*, ∀*j* ∈ *selCP*] and outputs non-propagated features, as proposed in [19].

The OCATA architecture for the C-band, however, is difficult to use directly in MB scenarios, since the ISRS impacts the channels differently, and therefore the number of pre-trained DNN models in the repository will be too high. Because of scalability reasons, the architecture of the novel OCATA-MB (see Figure 2b) considers a few RChs representing the specifics of different areas in the spectrum. Because OCATA models CP features propagation, the RChs are selected based on the different behavior of such features for the selected CPs. To decide which channels become RChs, a RCh selection procedure fairly selects them, making a balance between the complexity of the models and the accuracy of the final results. However, the target channel is not necessarily one of the selected RChs. Therefore, OCATA-MB includes a feature composition block to estimate the CP features for any channel in the C+L+S bands based on the output of the DNN models propagating the features of the RChs. The output of the feature composition block can then be used for QoT estimation, as in the classical OCATA architecture [18,19,20]. Note that the Constellation Reconstruction block is fed with the target channel to improve its accuracy in MB optical transmission.

### 2.3. QoT Estimation and Nonlinear Noise Mitigation

**Figure 3:**
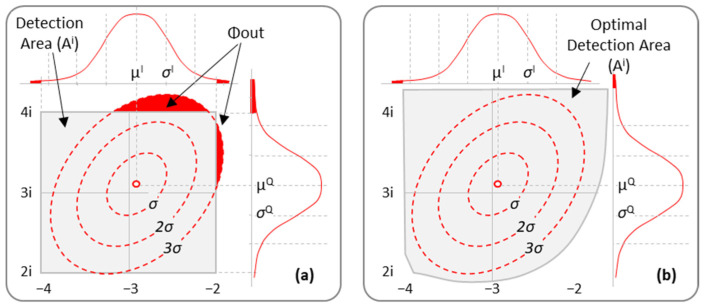
Example of $\Phi_{out}^{i}$ feature and regular (**a**) (reproduced from [20]) and optimized (**b**) detection area for CP *i* = [20].

The set of basic features introduced above can be extended. One parameter that can be computed from the basic features is $\Phi_{out}^{i}$ [20], which computes the probability of receiving a symbol originally sent as part of CP *i* out of the detection area assigned to that CP, represented as *A^i^*. $\Phi_{out}^{i}$ is computed under the assumption that the dispersion of symbols around CP *i* follows the bivariate Gaussian distribution characterized by *Y^i^*. For the sake of clarity, Figure 3a reproduces from [20] an example of $\Phi_{out}^{i}$ for CP [−3 + 3i]. The contours represent the different levels of the bivariate Gaussian distribution that characterize this CP for a given lightpath; univariate marginal distributions are provided for both the *I* and *Q* axes. The areas highlighted in red in both the bivariate and marginal distributions represent the region that falls out of *A^i^*, i.e., the square delimited by vertices (−4 + 4*i*) and (−2 + 2*i*). Hence, $\Phi_{out}^{i}$ is formally defined as follows:

(2) $$\Phi_{out}^{i}=1-\phi_{A}(i)$$

(3) $$\phi_{A}(i)=P\left(x\subset A^{i}|x\sim N\left(Y^{i}\right)\right)$$

Note that the estimated pre-FEC BER can be computed based on $\Phi_{out}^{i}$ for all the CPs, e.g., assuming equal probability of the symbols.

In view of Equations (2) and (3), the higher the *ϕ_A_*(*i*), the lower the pre-FEC BER will be. Therefore, a way to improve the QoT is by increasing *ϕ_A_*(*i*), which works for NLI noise mitigation. That can be implemented by changing the shape of *A^i^* in the Rx. To illustrate that, Figure 3b shows an adapted detection area for CP *i*. After modifying the detection area for this CP, the portion of the red shaded area is decreased, and, as a result, $\Phi_{out}^{i}$ reduces. However, finding the optimal *A^i^* for every CP so that the total Φ*_out_*, i.e., not only that for the CP, is minimized is a complex task. Hence, algorithmic solutions for computing a set of near-optimal detection areas in a reasonable time frame are required. By reducing the pre-FEC BER at the Rx, the reach of the lightpath can be extended; consequently, channel assignments that result in pre-FEC BER threshold violations and would be rejected, can now be possibly accepted. Note that the optimal *A^i^* for every CP is computed by OCATA-MB as part of the provisioning process, and a map is generated to be easily used by the Rx.

### 2.4. Lightpath Provisioning

**Figure 4:**
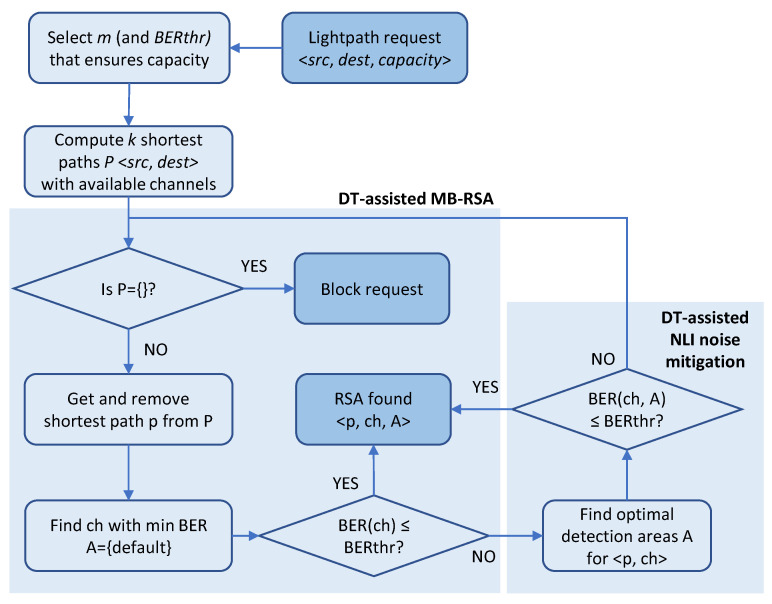
Proposed DT-assisted MB-RSA procedure.

Armed with the ability of estimating the QoT for different channel assignments in an efficient way, as well as with the possibility to improve the QoT of the signal by mitigating NLI noise, a DT-assisted MB-RSA algorithm that selects the path and the channel during lightpath provisioning can be devised. A simple flowchart is presented in Figure 4.

The procedure starts after receiving a request specifying the source and destination nodes and the required capacity. An MF and SR are selected to provide the required capacity, which determines the maximum pre-FEC BER that needs to be ensured. At this point, the procedure can be extended to consider multiple combinations of MF/SR, as well as multiple parallel lightpaths that provide the required capacity. Nonetheless, for the sake of simplicity, let us assume that just one lightpath with the best combination of MF/SR is considered, so *k* shortest paths between source and destination nodes with available channels are computed.

The procedure now iterates until a feasible solution is found, or otherwise the request is blocked. A set of available channels in the given route is computed. Then, the algorithm (*i*) determines whether there are available channels that can provide the required QoT given the characteristics of the route, specifically the total number of spans and their length; (*ii*) if some channel can meet the required QoT, then it finds the optimal channel assignment; (*iii*) otherwise, it selects the best channel assignment among the available channels, computes the optimal areas to mitigate the NLI noise as much as possible, and determines whether that solution would provide the required QoT.

The next section elaborates on the sketched OCATA-MB methodology and proposes algorithmic approaches.

## 3. Models and Algorithms

This section first describes how the pre-training process is carried out in OCATA-MB, which includes a pre-computation phase and therefore, is independent of the provisioning process. The initial (and subsequent if needed) OCATA-MB set-up might use data coming from different sources, including simulation, lab experiments, and operational ones. From them, the pre-training process populates an internal database with DNN models for optical links, with different characteristics in terms of the length and number of spans. Because the DNN link models need to be specific for each channel, RChs are selected and models are pre-trained (following [18,19,20]) only for those RChs, hence noticeably simplifying the pre-training process. The selected RChs are, as well, stored in an internal repository in OCATA-MB to be used during provisioning.

Next, the specific approach to be used during the provisioning process is presented. First, the features composition approach is presented. The proposed approach uses the features obtained after the features propagation process for the reference channels to estimate the features for a target channel, which is indeed a candidate for channel assignment. The procedure for the computation of optimal detection areas for NLI noise mitigation is proposed afterwards. Finally, our proposed OCATA-assisted MB-RSA algorithmic solution is described. Table 1 presents the notation used.

### 3.1. Selecting the Reference Channels

Let us first describe the selection of the set of RChs. It is worth noting that the *σ*^I^ and *σ*^Q^ features provide insights into the dispersion of the symbols around the mean of each CP. Therefore, we focus on analyzing these features to determine the smallest set of RChs that best represents the behavior of the complete set of channels in the spectrum.

The algorithm starts by retrieving a set of signal samples from the data repository, as well as the BER measured when those samples were obtained (line 1 in Algorithm 1). Next, features *Y* are computed using GMM fitting (line 2), which are subsequently filtered to select *σ^I^* and *σ^Q^* for the selected CPs (line 3).

The proposed approach relies on fitting piecewise linear functions representing the behavior of the *σ* features. The cut-points of the function are then candidates to become reference channels. The pseudocode is presented in Algorithm 1, which receives the order of MF, the set of selected CPs, and the largest admissible distance for the considered network (in km). It is clear that having a large number of reference channels will provide better accuracy and simplify the subsequent feature composition, However, that will increase the complexity of the DNN model pre-training process, Therefore, we target selecting just the smallest number of reference channels that provides good accuracy without adding excessive complexity to the features composition. In consequence, the algorithm analyzes the results of selecting a specific number of reference channels, which is increased until the desired accuracy is achieved (lines 4–11), or a maximum number of reference channels is exceeded (line 12). At every iteration, the algorithm fits each *σ* feature with the desired linear segments to obtain a list of cut-points, which are collected into a single set of candidate reference channels (lines 6–8). As a fitting function in line 7, we select a piecewise linear approach, due to its low complexity, easiness of interpretability, and ability to find the cut-points that allow the highest accuracy with simple linear segments, which extols the high relevance of the selected cut-points.

When all the features are processed, the set of candidate channels contains *i* clusters with possible reference points, so the next step (line 9) finds the centroids for each cluster, which are the candidate reference channels for this iteration. Then, the pre-FEC BER is estimated over these centroids (line 10) and compared with the real BER. In case the relative error is smaller than the target threshold, the candidates become the reference channels to be used and are returned (line 11). Otherwise, the process continues until the maximum admissible number of reference channels is reached (line 12).
**Algorithm 1** RCh Selection**INPUT**: *m*, *selCP*, *dmax*          **OUTPUT:** *RCh*   1:   2:   3:   4:   5:   6:   7:   8:   9: 10: 11: 12:[*X*], *BER_samples_* ← *getSamples*(*dmax*) [*Y*] ← *FeX*([*X*]) [*σ*] ← filterFeatures([*Y*], *selCP*, [*σ^I^*, *σ^Q^*]) **for each** *i* **in** 1..*MAX_RCh*
**do**      *RCh* ←Ø      **for each**
*σ*
**in** [*σ*] **do**             *cutPoints*←*fitPiecewiseLinearFunction*(*σ, i*)             *RCh* ← *RCh* U {*cutPoints*}      *cRCh*←findCentroids(*RCh*, *i*)      *BER_RC_*← computeBER([Y], *cRCh*)      **if** relativeError(*BER_RCh_*, *BER_samples_*) < ε **then return** *cRCh* **return** Ø

### 3.2. Feature Composition

The algorithm for feature composition uses the features of the two adjacent RChs to the target channel to linearly interpolate the features [*Y^i,ch^*] for that channel.

Algorithm 2 presents the pseudocode that receives as input the target channel and the set of features for the reference channels after propagation. The algorithm first finds the adjacent reference channels to the target channel (line 1 in Algorithm 2). Next, the features of the adjacent reference channels are selected from the complete set of features and used for linear fitting for each individual feature (lines 2–4). Finally, the features of the target channel are calculated by interpolation and finally returned (lines 5–6).
**Algorithm 2** Feature Composition**INPUT**: *ch,* [*Y^i,RCh^*]          **OUTPUT:** [*Y^i,ch^*]   1:   2:   3:   4:   5:   6:*lRCh*, *rRCh* ←*getAdjacentRChs*(*ch,* [*RCh*]) [*Y^i,lRCh^*] ←*getRChFeatures*(*lRCh*, [*Y^i,RCh^*]) [*Y^i,rRCh^*] ←*getRChFeatures*(*rRCh*, [*Y^i,RCh^*]) [*slope*]*,* [*intercept*] ← *getLineSegments*([*Y^i,lRCh^*], [*Y^i,rRCh^*]) [*Y^i,ch^*] ← *getFeaturesFromSegments*(*ch*, [*slope*]*,* [*intercept*]) **return** [*Y^i,ch^*]

### 3.3. Nonlinear Noise Mitigation

**Figure 5:**
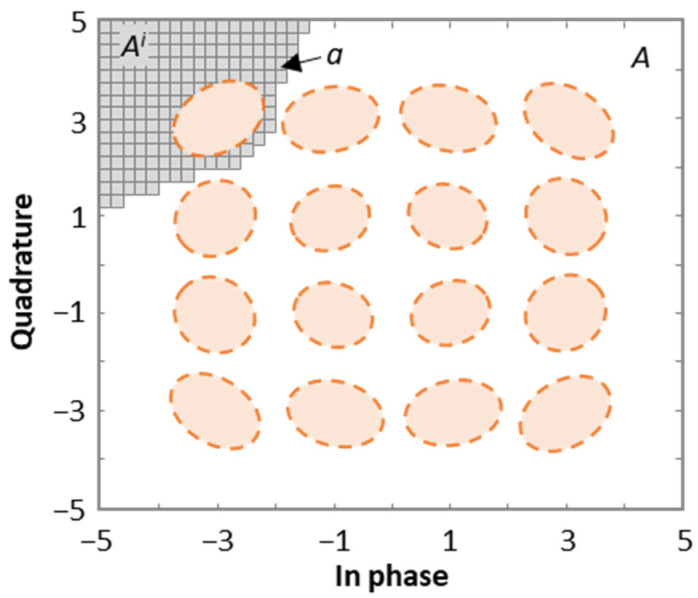
Definition grid for 16-QAM signal constellations.

NLI noise mitigation by optimizing the detection areas on the Rx side is addressed in this section. The proposed method computes near-optimal detection areas for the CPs and estimates the resulting pre-FEC BER. The method is based on dividing the whole coordinate IQ plane (*A*) into *k* small square areas *a* to create a grid. Figure 5 shows an example for a 16-QAM signal, where *A* is defined by corner points [5 + 5*i*] and [−5 − 5*i*]. The detection area for CP [3 + 3*i*] is shown as a set of small areas.

Then, the probability of receiving a symbol originally transmitted in CP *i* can be defined as Equation (4), which facilitates computing Φ*_A_* as Equation (5).

(4) $$\varphi_{a}\left(i\right)=P\left(x\subset a|x\sim N(Y^{i})\right)$$

(5) $$\phi_{A}(i)=\sum_{a\in A^{i}}\varphi_{a}\left(i\right)$$

With these definitions, the proposed method consists of assigning each of the small areas *a* to the detection area *A^i^* of the CP *i* with the highest probability. Algorithm 3 shows the pseudocode of the algorithm that receives features *Y*, the coordinate plane *A* divided into small areas and the number *m* of CPs in the signal, and computes a vector of detection areas [*A^i^*], where each area defines a subset of small areas of the whole coordinate plane.
**Algorithm 3** Compute Detection Areas**INPUT**: *Y*, *A*, *m*          **OUTPUT:** [*A^i^*]   1:   2:   3:   4:   5:   6:   7:   8:   9: 10:*A^i^* ← Ø, **for each** *i*:1..*m* **for each** *a* in *A* **do**      *a*.i ← 0      *a*.maxProb ← 0      **for**
*i*: 1..*m* **do**            **if** $\varphi_{a}(i)$> *a*.maxProb **then**                 *a*.maxProb ← $\varphi_{a}(i)$; see Equation (4)                 *a*.i ← *i*      *A^a.i^* ← *A^a.i^* ∪ *a* **return** [*A^i^*]

Vector [*A^i^*] is then transformed into matrix *D*, where every position in the matrix identifies the CP *i* for decoding the symbols detected in the related small area *a*. Matrix *D* is sent to the Rx as part of the lightpath provisioning phase, so the Rx can now easily decode every received symbol *x* using Equations (6)–(8).

(6) $$colIdx=\left\lfloor \frac{x^{I}}{\delta }\right\rfloor +\frac{\sqrt{k}}{2}$$

(7) $$rowIdx=\left\lfloor -\frac{x^{Q}}{\delta }\right\rfloor +\frac{\sqrt{k}}{2}$$

(8) i = D[rowIdx][colIdx]

### 3.4. Lightpath Provisioning

This subsection proposes a heuristic algorithm to solve the on-line MB-RSA problem for lightpath provisioning. The pseudocode is presented in Algorithm 4.
**Algorithm 4** OCATA-Assisted on-line MB-RSA**INPUT**: *G*(*N, E*), <*src*, *dest, m*, *BER_thr_*>, *OCATA_MB***OUTPUT:** *p*, *ch,* [*A^i^*]   1:*P* ← *kSP(src*, *dest)*   2: **while** *P* ≠ Ø **do**
   3:       *p* ← *removeFirst*(*P*)   4:       *Ch* ← *getAvailableChannels*(*p*)   5:       *ch*, [*A^i^*] ← *OCATA_MB.*selectCh(*p*, *Ch*, *m*, *BER_thr_*) (Algorithm 5)   6:       **if** *ch* is not **None then return** <*p*, *ch,* [*A^i^*]>   7: **return None**

The heuristic receives a connected graph *G*, a connection request, and the interface to OCATA-MB. The graph *G* includes the set *N* with the optical nodes and the set *E* with the optical links connecting two nodes, where each link *e* specifies the status of the channels in every band. The connection request is specified by the pair of source and destination nodes, the order of modulation format *m*, and the BER threshold to be ensured. The algorithm first computes a set of shortest routes in terms of total distance on the network graph (line 1 in Algorithm 4). Next, the algorithm explores every route in the list to determine the optimal channel (lines 2–6). Then, for every route *p*, the set of channels that are available in all the links is computed (line 4). The algorithm is assisted by OCATA-MB for the selection of the optimal channel and to compute the optimal detection areas for NLI noise mitigation if needed (line 5). The route and channel, together with the detection areas when NLI noise has to be mitigated to meet the requested QoT, are returned (line 6). Otherwise, the next route is explored until the list is exhausted, in which case the algorithm returns no solution found (line 7).

Algorithm 5 presents the pseudocode for channel selection. The algorithm receives the selected route, the set of available channels, the number *m* of CPs, and the BER threshold to be assured, and it returns the selected channel and the detection areas in case NLI noise mitigation is needed. The algorithm first retrieves the list of pre-computed reference channels from an internal repository (line 1 in Algorithm 5) and composes an end-to-end DNN model for the selected route for each of the reference channels (line 2) by concatenating individual link models [19]. Next, input samples *X* are randomly generated for each reference channel, and features *Y* are computed using GMM fitting; only the features for the selected CPs are used for propagation, being the features for the other CPs discarded (line 3). The input features are then propagated using the end-to-end DNNs models for each of the reference channels, which produces a set of output features characterizing the signal at the Rx (line 4).
**Algorithm 5** OCATA-MB Channel Selection**INPUT**: *p, Ch, m, BER_thr_*         **OUTPUT:** *ch,* [*A^i^*]   1: [RCh] ← getRCh()   2: [*M*] ← getModelsforRoute(*p,* [*RCh*])   3: [*Y^i,RCh^*] ← generateInputFeatures([*RCh*])   4: [*Y^i,RCh^_out_*] ←*propagate*([*Y^i,RCh^*]*,* [*M*])   5: *Ch_aux_* ← *Ch*   6: *BERvsCh* ← *getBERCurve*(*p*)   7: **while**
*Ch* ≠ Ø **do**   8:       *ch*, *BER*_max_ ← *getChWithMaxBER*(*BERvsCh*, *Ch*)   9:       **if**
*BER*_max_ > *BER*_th_ **then** 10:             *Ch* ← *Ch* \ {*ch*} 11:             **continue** 12:       [*Y^i,ch^_out_*]← featureComposition([*Y^i,RCh^_out_*], *ch*) (Algorithm 2) 13:       *Y^ch^_out_* ← constReconstruction([*Y^i,ch^_out_*]) 14:       *BER^ch^* ← estimateBER(*Y^ch^_out_*) 15:       **if** *BER^ch^* ≤ *BER*_th_ **then return** <*ch, -*> 16:       *Ch* ← *Ch* \ {*ch*} 17: *ch* ← *getChWithMinBER*(*BERvsCh, Ch_aux_*) 18: [*Y^i,ch^_out_*]← featureComposition([*Y^i,RCh^_out_*], *ch*) (Algorithm 2) 19: *Y^ch^_out_* ← constReconstruction([*Y^i,ch^_out_*]) 20: [*A^i^*], *BER* ←computeNLIMitigation(*Y^ch^_out_*, *m*) (Algorithm 6) 21: **if**
*BER* ≤ *BER*_th_ **then return** *<ch*, [*A^i^*]*>* 22: **return None**

The output features for the reference channels are used to explore available channels in terms of estimated BER. However, because the list of available channels might be extensive, pre-computed pre-FEC BER vs. channel curves, similar to those in Figure 1b, are used to accelerate finding the channels that potentially would provide minimum and maximum BER for a given set. Then, the most appropriate curve for the selected route is retrieved (line 6). The available channels are sequentially explored (lines 7–16) by selecting the one that potentially would provide the maximum BER and checking that the BER could be under the given threshold (lines 8–9). If the anticipated BER is above the threshold, that channel is discarded and the process restarts (lines 10–11). Otherwise, the channel is a candidate to support the lightpath, and its BER is accurately estimated. To that end, the specific features for the candidate channel are computed using the features composition algorithm, and the features of the non-propagated CPs are computed (lines 12–13). With the features of the entire expected optical signal for the candidate path, the pre-FEC BER is estimated (line 14), and the candidate channel is returned in case the estimated value is found to be under the threshold (line 15). Otherwise, the channel is discarded, and the process continues with the rest of the available channels (line 16).

In the case that none of the available channels can provide the requested BER, the application of NLI noise mitigation is considered (lines 17–21). To that end, the available channel with the minimum BER is selected using the pre-computed pre-FEC BER vs. channel curve (line 17), and the features for that channel are computed from those of the reference channels, as well as the features of the non-propagated CPs (lines 18–19). The optimal detection areas for the expected signal are then computed using an algorithm that also returns the estimated BER after NLI noise mitigation (line 20). In the case that the resulting BER is under the threshold, the channel, together with the computed detection areas, is returned (line 21); otherwise, none of the available channels has been found to guarantee the BER threshold (line 22).

Finally, Algorithm 6 presents the pseudocode for NLI noise mitigation that receives as input the expected features of the signal at the Rx and the number *m* of CPs in the signal and computes the detection areas, as well as the BER estimated based on probabilities $\Phi_{out}^{i}$ computed on the corresponding detection area *A^i^* for each CP. The algorithm first creates a grid by dividing the coordinate plane into small non-overlapping squares (*a*), with width and height equal to *δ*, and the detection areas are obtained using Algorithm 3 (lines 1–2 in Algorithm 6). Next, Φ*_out_* is estimated by computing $\Phi_{out}^{i}$ for each CP *i* on the defined grid and averaging for the number of CPs to obtain the symbol error rate (lines 3–9). Finally, the BER is estimated and returned together with the computed detection areas (line 10).
**Algorithm 6** Nonlinear Mitigation**INPUT**: *Y, m*          **OUTPUT:** [*A^i^*], *BER*   1: *A*←CreateGrid()   2: [*A^i^*] ← ComputeDetectionAreas(*Y*, *A*, *m*) (Algorithm 3)   3: Φ*_out_*←0   4: **for**
*i* = 1..*m*
**do**   5:       Φ*_A_*(*i*) ← 0   6:       **for each**
*a* in *A^i^*
**do**   7:             Φ*_A_*(*i*) ← Φ*_A_*(*i*) *+ a*.maxProb   8:       $\Phi_{out}^{i}$←1 − Φ*_A_*(*i*)   9:       Φ*_out_* ← Φ*_out_ +* $\Phi_{out}^{i}$*/m* 10: **return** [*A^i^*], Φ*_out_*/log_2_ *m*

## 4. Simulation Results

In this section, we evaluate the OCATA-MB digital twin to support MB lightpath provisioning, as well as the proposed on-line MB-RSA algorithm. First, the simulation environment used to generate the datasets needed for training DNN models is presented, and the architecture of the DNN link models is defined. Next, the RChs are selected and used for the feature composition. Then, the nonlinear mitigation technique is evaluated as stand-alone and, finally, used as part of the MB-RSA algorithm.

### 4.1. MB Optical System Simulation and Modeling

A MATLAB-based simulator of a coherent WDM system developed in [22] was used to generate IQ constellations for 16QAM@32GBd signals shaped by a root-raised cosine filter with a 0.06 roll-off factor. For the C+L+S system, we considered 337 optical channels with 50 GHz channel spacing for full spectrum usage. We assumed a pre-FEC BER threshold equal to 10^−2^ in all the cases. Table 2 summarizes the configurations used in the MB transmission simulator.

Pseudorandom binary sequences of length 2^16^ were used as the input of every channel. The signal was propagated through spans of standard single-mode fiber, ranging from 70 to 100 km, with a launch power of 0 dBm. Gain flattening filters were used at the end of each fiber span to compensate for the power tilt induced by the ISRS effect. The attenuation factor, chromatic dispersion, and nonlinear (gamma) parameters varied with frequency. The fractional contribution of the delayed Raman response was 0.245 [29]. Propagation in optical fiber was modeled by solving the nonlinear Schrödinger equation using the Runge-Kutta method (see [22]). EDFAs and TDFAs were modeled as ideal OAs characterized by a single gain and noise figures. Note that such simplification is intended to clearly show the differentiated behavior of the channels in the different transmission bands. An adaptive step-size algorithm was also included to further reduce the computation time required by the Runge–Kutta method. Finally, at the Rx, a DSP block performed ideal chromatic dispersion compensation and phase recovery.

The dataset was populated with a total of 1000 samples, which were used for training, validating, and testing the DNN link models [30]. The DNN link models were characterized by: (*i*) 20 inputs for the features of the four selected CPs; (*ii*) two hidden layers with 12 neurons and *tanh* (·) activation function; and (*iii*) 20 outputs for the propagated features. A specific DNN link model was trained for each different RCh and configuration of the link, i.e., the number of spans and span length (in steps of 10 km).

### 4.2. Reference Channels Selection

**Figure 6:**
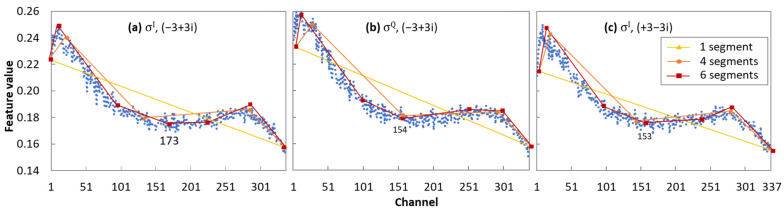
Value of selected σ features and CPs vs. channel index and piecewise linear fitting with 1, 4, and 6 segments. (**a**) *σ^I^*, (−3 + 3i), (**b**) *σ^Q^*, (−3 + 3i), and (**c**) *σ^I^*, (3 − 3i).

Let us first select the RChs that will be afterwards used during the lightpath provisioning process. Figure 6 shows the value for every channel of some *σ* features belonging to two exterior CPs.

We observe that a piecewise linear approach with six segments results in relatively accurate models. Note that six segments result in seven different RChs. However, the location of the cut-points is different for each individual feature, as well as for the different link configurations. For example, the fourth cut-point ranges from 153 to 174 in the plots in Figure 6. Therefore, Algorithm 1 finds the centroid among the values for the same cut-point index from the different *σ* features and link configurations.

**Figure 7:**
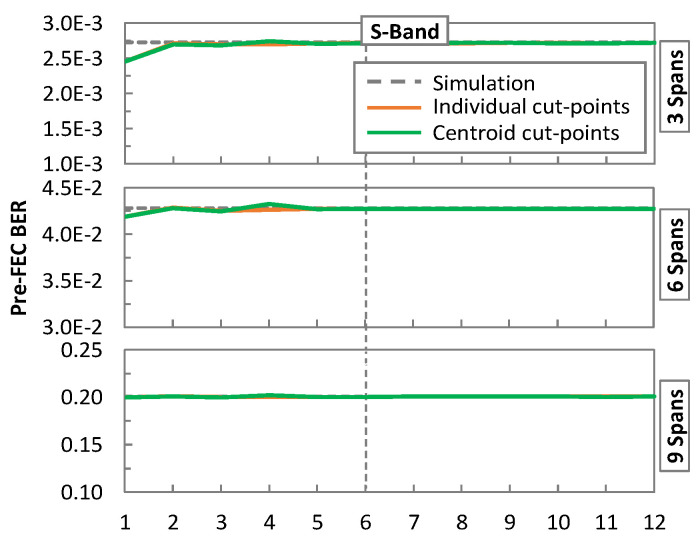

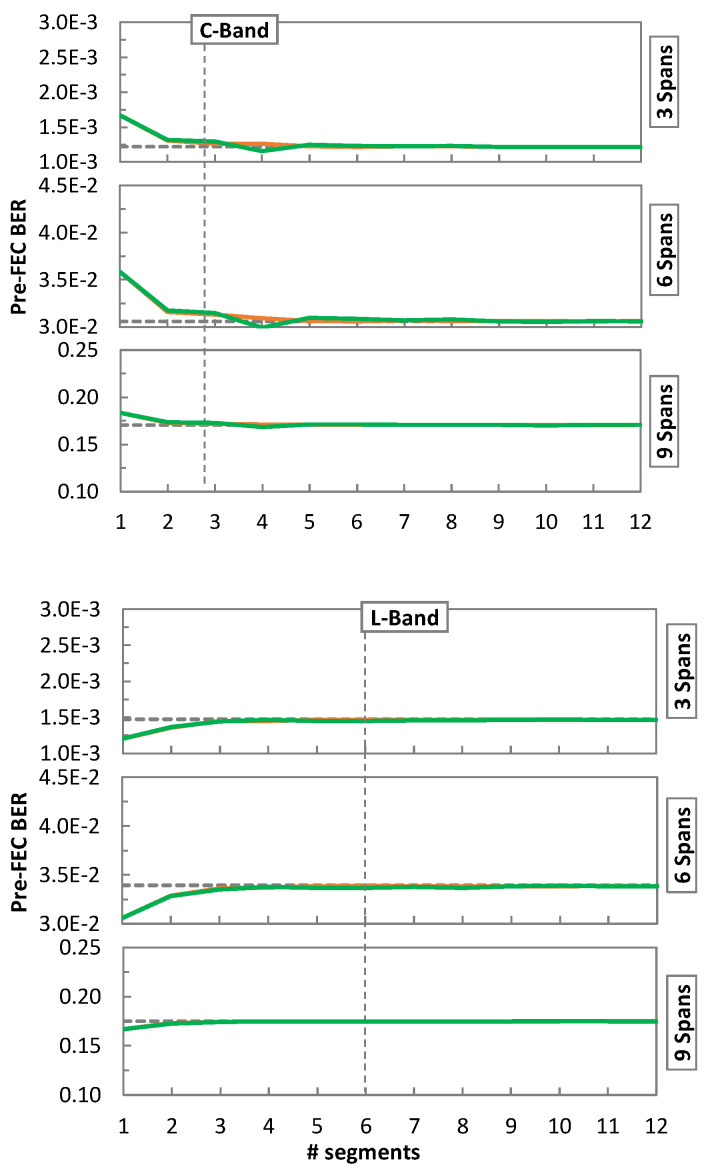
Pre-FEC BER vs. number of piecewise linear segments.

Figure 7 shows the results of an extensive study using the generated datasets, where pre-FEC BER is shown for links with three, six, and nine spans where the pre-FEC BER is estimated from: (i) the results of the simulation; (ii) using the individual cut-points for every *σ* feature; and (iii) using the computed centroids for each cut-point. The averaged values for the channels in each band are shown in separate graphs. We observe the accuracy of pre-FEC BER estimation from the features of the signal and conclude that fitting six linear segments provides reasonable accuracy with moderated complexity.

Finally, the selected RChs are: channels 1, 20, and 97 in the S-band, channel 154 in the C-band, and channels 225, 304, and 337 in the L-band.

### 4.3. Feature Composition

Let us focus now on the composition of the features. Recall that the features of the RChs after propagation through the set of DNN link models for the given route are already available, and Algorithm 2 is used to estimate those for a target channel.

**Figure 8:**
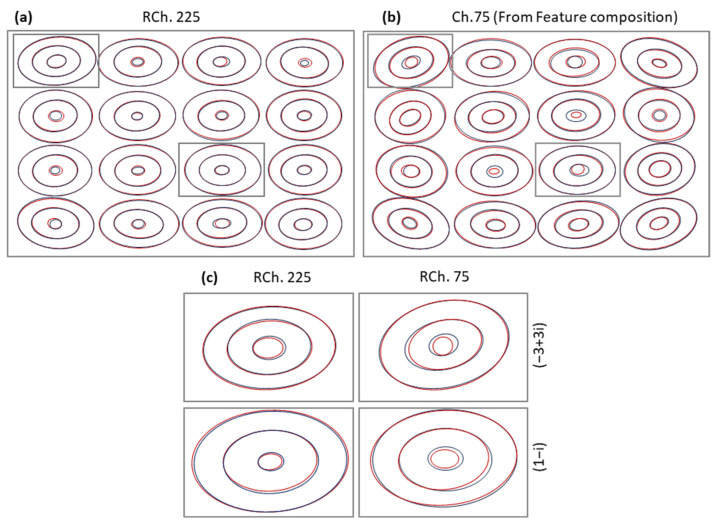
Reconstructed constellations for an RCh (**a**) and a non-RCh (**b**). Details of two CPs, one exterior and one interior (**c**).

Figure 8 shows the contour plots for RCh 225 (Figure 8a) and the non-propagated channel 75 (Figure 8b) after signal propagation along nine spans. Figure 8c focuses on the two propagated CPs (one interior and one exterior) for RCh 225 and the composed ones for channel 75. In both cases, we observe high accuracy in the channels estimated features (in red) w.r.t. the ones obtained from the simulator (in black).

**Figure 9:**
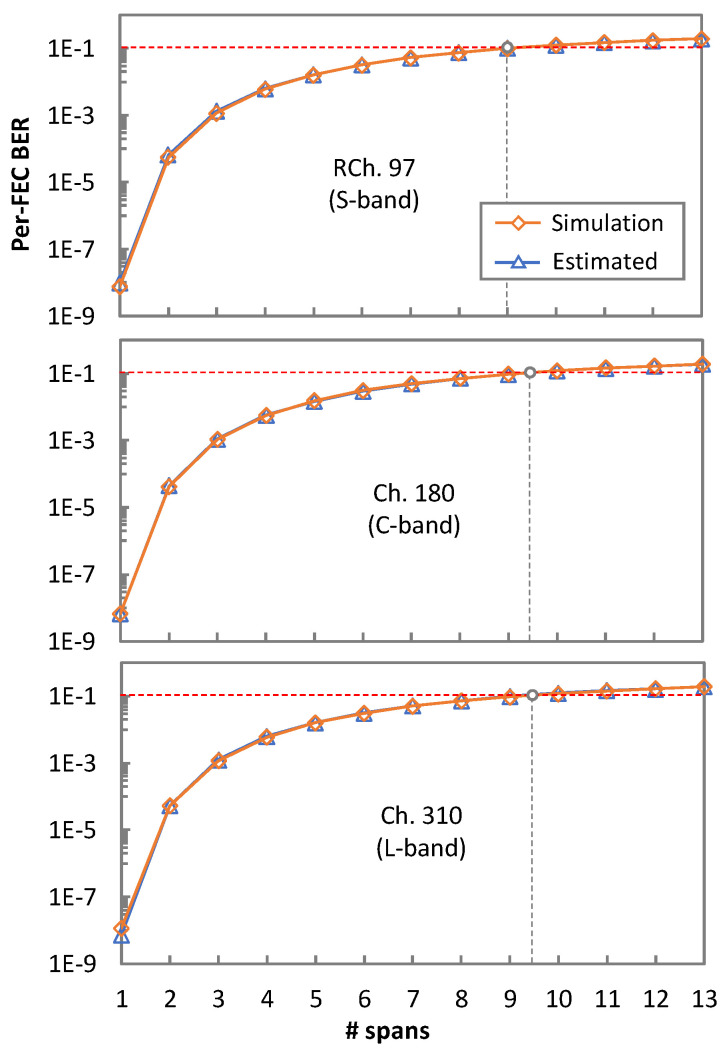
Evolution of pre-FEC BER as a function of the number of spans for several channels.

To evaluate the accuracy of the QoT estimation, Figure 9 compares the pre-FEC BER calculated by numerical simulation with the one estimated using Algorithm 6 without NLI noise mitigation, i.e., assuming square boundaries for the detection areas. The results obtained for RCh 97 and two non-propagated ones, i.e., ch. 180 and ch. 310, are depicted for different transmission distances. We observe that the pre-FEC BER estimation for the three channels is very accurate, in line with the results in [20] for the C-band.

### 4.4. Nonlinear Mitigation

**Figure 10:**
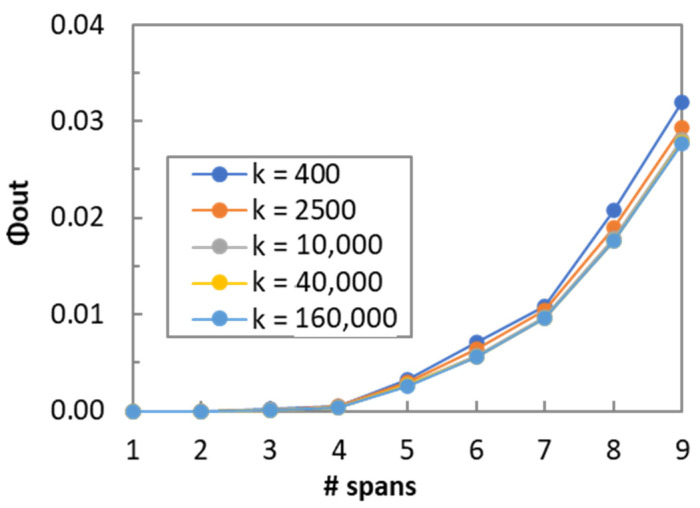
Estimated pre-FEC BER for different number of areas.

Let us now analyze the number of areas k that need to be considered in Algorithm 6 to obtain accurate Φ_out_ computations. Figure 10 illustrates the convergence of Φ_out_ with *k* for channel 337; similar results can be obtained for the other channels. We observe that a very small error is obtained when 10,000 or more areas are considered, which greatly limits the time of Algorithm 3 to compute the detection areas.

**Figure 11:**
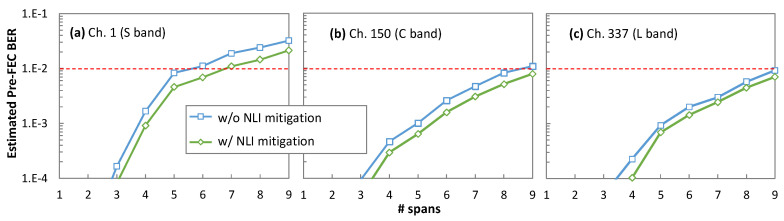
Real and estimated pre-FEC BER with squared and optimized detection areas vs. # spans for ch. 1 (**a**), 150 (**b**), and 337 (**c**).

**Figure 12:**
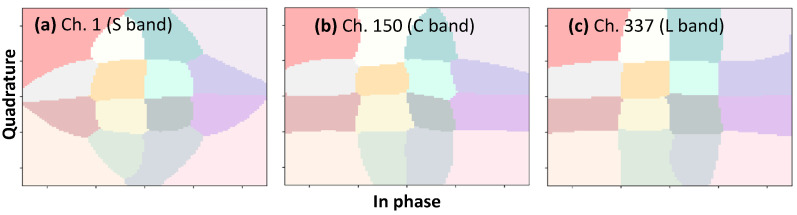
Optimal detection areas (k = 10,000) for QAM-16 signals for five spans. (**a**) Ch. 1 in the S band, (**b**) Ch. 150 in the C band, and (**c**) Ch. 337 in the L band.

Figure 11 shows the estimated pre-FEC BER with and without nonlinear mitigation with the detection areas optimized using Algorithm 6 for three channels, each one from a different band, as a function of the number of fiber spans, when fixing *k* = 10000. We observe an improvement in the resulting pre-FEC BER in all three channels independently of the transmission distance. In particular, we observe that the maximum transmission distance is highly dependent on the selected channel, which stresses the application of OCATA-MB during the lightpath provisioning phase to select a channel that can ensure that the pre-FEC BER threshold is not exceeded. In addition, by configuring the optimal detection areas in the Rx, the maximum achievable transmission distance is effectively extended. Indeed, Figure 11 shows that the reach of Channel 1 can be extended by one additional span without exceeding the pre-FEC BER threshold when nonlinear mitigation is implemented. Similar conclusions can be drawn for Channels 150 and 337. For illustrative purposes, Figure 12 shows the optimized detection areas for Channels 1, 150, and 337 after transmission along six spans. We observe different shapes of the detection areas for the different channels needed to mitigate the different impact of NLI noise, mainly due to SRS.

### 4.5. Lightpath Provisioning

**Figure 13:**
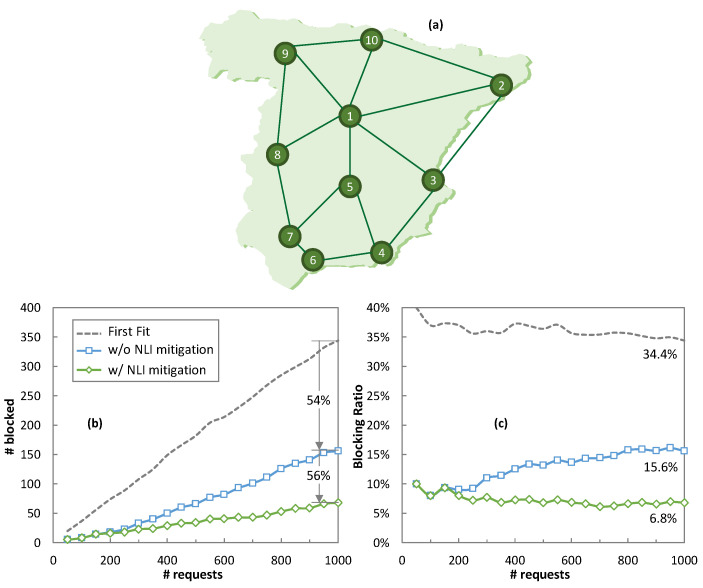
Spanish core optical network topology (**a**). Number of demands blocked (**b**) and blocking ratio evolution (**c**) vs. demand number.

Finally, let us evaluate the performance of the OCATA-assisted on-line MB-RSA algorithm defined in Algorithm 4. To this end, a simulation environment was implemented in Python, and the Spanish 10-node core network topology (Figure 13a) with links with various configurations was assumed. In addition, to evaluate the performance of the NLI noise mitigation strategy, Algorithm 4 was used with and without NLI noise mitigation (i.e., with and without executing lines 17–21). For benchmarking purposes, the traditional first-fit channel assignment with QoT assurance was considered, where QoT was estimated for the selected channel, and the request was rejected in case the required QoT was not met for that channel.

The simulation assumed a static traffic model, i.e., the simulation started with no traffic on the network, and connection requests were served sequentially between randomly chosen source and destination nodes. Once a connection was successfully provisioned on the network, it stayed without being changed or removed from the network. For the sake of simplicity, a set of 1000 connection requests was generated beforehand, checking that the shortest route could provide the required QoT for at least one channel assignment. The simulator was then run for every considered approach with the same pre-generated list of connection requests. The number of shortest routes between each pair of nodes was limited to three in all the cases.

Figure 13b shows the number of requests blocked, whereas Figure 13c depicts the blocking ratio as a function of the number of connection requests. We observe that the first-fit approach provided the highest number of connection requests blocked in all the cases because the channel selected could not provide acceptable QoT, i.e., blocking was not a consequence of a lack of available channels in the selected route. The final blocking ratio was as high as 34% after processing 1000 connection requests. The number of requests blocked was highly reduced by 54% (from 344 down to 156) when the OCATA-assisted on-line MB-RSA algorithm (Algorithm 4) was applied to find the best channel that could provide the needed QoT. In this case, the final blocking ratio reduced to 15.6%. Finally, when the NLI noise mitigation technique was also implemented, the number of requests blocked remarkably reduced by an additional 56% (from 156 down to 68), with a final blocking ratio of just 6.8%. Hence, the total blocking reduction when the NLI noise mitigation technique is implemented is over 80%.

## 5. Concluding Remarks

A digital twin-assisted lightpath provisioning approach has been proposed for providing route and channel assignment that can provide the required QoT in multiband optical networks. The particular impact of nonlinear impairments on each optical channel in the spectrum of the C+L+S transmission bands makes channel assignment a non-trivial problem, with a huge impact on the network resource utilization and thus on the capability of the network to provide optical connections. In view of that, the OCATA-MB time domain digital twin is proposed, not only to provide QoT estimation of a given route and a channel assignment, but also to actively participate in the channel assignment itself.

OCATA includes DNNs modeling optical signal propagation in optical networks. Because of the different impact of nonlinear noise on the different channels and the large number of channels available in the C+L+S MB transmission systems, OCATA-MB includes a special methodology that defines reference channels to reduce the number of DNN models and a feature composition block that provides the features for any target channel by interpolating those of the two adjacent reference channels. It is worth noting that the number of considered reference channels plays an important role in the accuracy of the procedure, as well in the scalability of the OCATA-MB methodology. Therefore, an algorithm is proposed to define the number and identify the reference channels to be used for DNN modeling. Finally, nonlinear mitigation is proposed to improve the QoT of optical connections by optimizing the detection areas in the Rx. NLI noise mitigation can then be used in the case that a lightpath cannot be provisioned because none of the available channels can provide the required QoT, and therefore such nonlinear mitigation is a valuable tool to reduce connection blocking.

The performance of the proposed OCATA-MB methodology was assessed using numerical simulation. The results showed remarkable accuracy in the estimation of the features of the reference channels after they were propagated by the DNN models, as well as for the non-reference channels whose features were estimated from the RChs. The accuracy of the proposed approach was demonstrated when estimating the QoT, specifically the pre-FEC BER. The performance of the nonlinear noise mitigation technique was also assessed, and the results showed an important improvement in the QoT for different channel assignments. Finally, the performance of the proposed OCATA-MB-assisted lightpath provisioning approach was evaluated, and the results showed an over 50% improvement in terms of the blocking ratio w.r.t. a traditional first-fit algorithm with QoT estimation when NLI noise mitigation was not implemented, and an additional 50% of improvement when the NLI noise mitigation was applied. The obtained results clearly show the usefulness of the proposed methodology.

## Figures and Tables

**Table 1:** Notation.

General	
*X*	Set of symbols, index *x*.
*Y^i^*	Features of CP
*m*	Order of modulation format and number of CPs
*selCP*	Set of selected CPs to be propagated by the OCATA DNN models
**NLI noise mitigation**	
*A*	The whole coordinate plane, which is divided into small squares *a*.
*A^i^*	Detection area for CP *i*.
*k*	Number of squares areas *a*, each of size *δ* × *δ*
*D*	Square matrix of size sqrt(*k*) × sqrt(*k*) for symbol decoding.
*ϕ_A_*(*i*)	Probability that a symbol from CP *i* is received inside the detection area *A^i^*.
$\Phi_{out}^{i}$	Complementary probability of *ϕ_A_*(*i*).
*φ_a_*(*i*)	Probability of a symbol from CP *i* is received inside the small square *a* ∈ *A^i^*.

**Table 2:** Considered MB optical systems.

	S-Band	C-Band	L-Band
Wavelength range [nm]	1481.7–1530.1	1530.1–1564.8	1564.8–1616.7
Bandwidth [THz]	6.19	4.35	5.76
Num of Channels (Ids)	128 (1–128)	87 (129–215)	122 (216–337)
Type of Amplifier	TDFA	EDFA	EDFA
Amplifier Noise Figure	5		
Nonlinear coefficient Dispersion, Attenuation	Vary with frequency [5]		
Launch Power	0 dBm		

## Data Availability

Data is available in [30].
